# A Unique Pool of Compatible Solutes on *Rhodopirellula baltica*, Member of the Deep-Branching Phylum *Planctomycetes*

**DOI:** 10.1371/journal.pone.0068289

**Published:** 2013-06-27

**Authors:** Ana Filipa d’Avó, Sofia Cunha, Ana Mingote, Pedro Lamosa, Milton S. da Costa, Joana Costa

**Affiliations:** 1 Center for Neurosciences and Cellular Biology, University of Coimbra, Coimbra, Portugal; 2 Instituto de Tecnologia Química e Biológica, Universidade Nova de Lisboa, Oeiras, Portugal; 3 Centro de Ressonância Magnética António Xavier, Instituto de Tecnologia Química e Biológica, Universidade Nova de Lisboa, Oeiras, Portugal; 4 Department of Life Sciences, University of Coimbra, Coimbra, Portugal; University of Groningen, The Netherlands

## Abstract

The intracellular accumulation of small organic solutes was described in the marine bacterium *Rhodopirellula baltica*, which belongs to the globally distributed phylum *Planctomycetes* whose members exhibit an intriguing lifestyle and cell morphology. Sucrose, α-glutamate, trehalose and mannosylglucosylglycerate (MGG) are the main solutes involved in the osmoadaptation of *R. baltica*. The ratio and total intracellular organic solutes varied significantly in response to an increase in salinity, temperature and nitrogen content. *R. baltica* displayed an initial response to both osmotic and thermal stresses that includes α-glutamate accumulation. This trend was followed by a rather unique and complex osmoadaptation mechanism characterized by a dual response to sub-optimal and supra-optimal salinities. A reduction in the salinity to sub-optimal conditions led primarily to the accumulation of trehalose. In contrast, *R. baltica* responded to salt stress mostly by increasing the intracellular levels of sucrose. The switch between the accumulation of trehalose and sucrose was by far the most significant effect caused by increasing the salt levels of the medium. Additionally, MGG accumulation was found to be salt- as well as nitrogen-dependent. MGG accumulation was regulated by nitrogen levels replacing α-glutamate as a K^+^ counterion in nitrogen-poor environments. This is the first report of the accumulation of compatible solutes in the phylum *Planctomycetes* and of the MGG accumulation in a mesophilic organism.

## Introduction

A large number of microorganisms rely exclusively on the accumulation of low-molecular-weight organic compounds, designated compatible solutes, for osmoadaptation, indicating that this is a very successful strategy [1]. Compatible solutes can be uptaken from the environment or synthesized *de novo* to fulfill the specific requirements of the organism. Initially, these compatible solutes, also named osmolytes, were associated with the need to reach an osmotic equilibrium, but are now viewed as very versatile protectors of cell components against a number of stress agents [2]. Osmolytes are normally neutral or zwitterionic compounds under the conditions experienced inside the cells and belong to a few restricted chemical categories. Moreover, some compatible solutes are widespread in nature whereas others are restricted to a few groups of organisms [1].

An increase in the intracellular potassium during low-level osmoadaptation is observed, contributing to the osmotic balance across the membrane and to the stabilization of the cellular turgor pressure. The resulting excessive positive charge is counterbalanced by the accumulation of α-glutamate. Overall, the accumulation of α-glutamate, a universal negatively charged compatible solute, reaches a physiological plateau prior to the activation of the osmoadaptive phenomena to higher salt stress [3], [4].

Among the sugar and sugar derivative compatible solutes, trehalose is a widespread disaccharide occurring in prokaryotic and eukaryotic organisms [3]. Sucrose is found in several plants, being later found in unicellular eukaryotic organisms, as well as in freshwater and marine cyanobacteria and in two species of halotolerant methanotrophs [5], [6]. Within marine microorganisms, namely in cyanobacteria, the accumulation of sucrose and trehalose as primary compatible solutes has been associated with a rather low salt tolerance [7]. Additionally, evidence suggests that these osmolytes have more intricate functions, acting as general stress protectors [7].

The sugar-glycerate derivative compatible solute, mannosylglucosylglycerate (MGG) has only been found in two thermophilic organisms, *Petrotoga miotherma* and *Petrotoga mobilis* [8], [9]. In the slightly halophilic bacterium *P. miotherma*, MGG was reported as the principal compatible solute accumulated during sub-optimal salinities, while in *Petrotoga mobilis* MGG was mainly accumulated during growth at supra-optimal salinities and temperatures [8], [9]. Curiously, α-glutamate was the major solute used by *P. miotherma* for osmoprotection at supra-optimal salinity [9].

The phylum *Planctomycetes* is a broadly distributed group of bacteria whose members can be found in terrestrial, freshwater and more abundantly in marine habitats [10]–[12]. These organisms are an unusual, but widely distributed group of bacteria which are proving to have increasing relevance in areas of research, such as microbial ecology and evolution, oceanography and wastewater treatment [13], [14]. *Planctomycetes* are unique among the bacteria because their cell walls do not contain peptidoglycan and they show a unique cell compartmentalization with a structure that is analogous to a eukaryotic nucleus [15]. From a phylogenetic perspective, the *Planctomycetes* form an independent, monophyletic phylum of the domain Bacteria that has been recently suggested to be the deepest branching bacterial phylum [14].

*Rhodopirellula baltica* is a marine representative of this phylum [16]. Marine microorganisms, like *R. baltica*, are exposed to rapidly changing environmental conditions such as, temperature, radiation, oxygen concentration, nitrogen availability and especially salinity. Concomitantly, alterations in nitrogen content also represent stressful conditions since most bacterial growth is usually limited by the amount of available nitrogen in the open ocean [17]. Usually, sudden changes on these environmental conditions induce a stress response that enables organisms to protect vital processes and to adapt to the new condition. Indeed, it has been reported that the exposure of *R. baltica* cells to high salinities and temperature resulted in the modulation of genes coding for compatible solutes, ion transporters and morphology. In fact, over 3000 of the 7325 genes were affected by temperature and/or salinity changes [18].

In this study we examined the intracellular pool of compatible solutes in *R. baltica* in response to growth phase, salinity, temperature and nitrogen-depletion to obtain insights into the physiologic role of each osmolyte. Moreover, we also tested the outcome of thermal and osmotic shocks under nitrogen-limiting conditions. To our knowledge, this is the first description of the compatible solutes accumulation spectrum in a member of the peculiar phylum *Planctomycetes*.

## Materials and Methods

### Strains and Culture Conditions

*Rhodopirellula baltica* (DSM 10527) was obtained from the Deutsche Sammlung von Mikroorganismen und Zellkulturen, Braunschweig. *R. baltica* was routinely maintained at 25°C in modified M13a medium [19]–[21] containing per liter: glucose, 1.8 g; ammonium sulfate, 1 g; 500 ml of Artificial Sea Water (100%); 0.1 M phosphate buffer (pH 7.5 for liquid medium and pH 8.5 for solid medium); 10 ml of vitamin solution and 20 ml of the Hutner’s basal salts. The vitamin solution contained per liter: vitamin B_12_, 0.2 mg; biotin, 4 mg; thiamine-HCl×2 H_2_O, 10 mg; Ca-pantothenate, 10 mg; folic acid, 1 mg; riboflavin, 10 mg; nicotinamide, 10 mg; p-aminobenzoic acid, 10 mg; and pyridoxine hydrochloride, 10 mg. The Hutner’s salts solution contained per liter: nitrilotriacetic acid, 10 g; MgSO_4_×7 H_2_O, 29.7 g; CaCl_2_×2 H_2_O, 3.335 g; (NH_4_)_6_MoO_7_O_24_×4 H_2_O, 9.25 mg; FeSO_4_×7 H_2_O, 99 mg; and 50 ml of “Metals 44” solution. The “Metals 44” solution contained per liter: Na-EDTA, 250 mg; ZnSO_4_×7 H_2_O, 1095 mg; FeSO_4_×7 H_2_O, 500 mg; MnSO_4_×H_2_O, 154 mg; CuSO_4_×5 H_2_O, 39.2 mg; Co(NO_3_)_2_×6 H_2_O, 24.8 mg; and Na_2_B_4_O_7_×10 H_2_O, 17.7 mg. The Artifical Sea Water (100%) contained per liter: NaCl, 23.47 g; Na_2_SO_4,_ 3.92 g; MgCl_2_×6 H_2_O, 10.64 g; CaCl_2,_ 1.10 g; NaHCO_3,_ 192.0 mg; KCl, 664.0 mg; KBr, 96.0 mg; H_3_BO_3,_ 26.0 mg; SrCl_2,_ 24.0 mg; and NaF, 3.0 mg [22]. This modified M13a medium was used has standard medium for control purposes in all experiments and called N^+^. Additionally, ampicilin was added to a final concentration of 0.1 mg/ml to both liquid and solid media to avoid contaminations.

To study the compatible solute content during growth, cells were grown in Erlenmeyer flasks (500 ml) in an orbital shaker under distinct conditions, described below. Cultures were harvested at appropriate time intervals, washed twice with a NaCl and MgCl_2_.6H_2_O solution identical to the concentration of the growth medium.

### Extraction of Intracellular Organic Solutes

Organic solutes were extracted twice with boiling 80% ethanol, as previously described [23]. The solvent from the combined supernatants was removed by rotary evaporation, and the residue was freeze-dried. The dry residue was suspended in distilled water and extracted twice with a mixture of water-chloroform (2∶1) to clean the lipid components. After centrifugation (3000×g and 4°C for 10 min), the aqueous phase was lyophilized and the residue was dissolved in ^2^H_2_O for magnetic resonance (NMR) analysis. The cell protein content was determined by the Bradford assay [24] after cell disruption by sonication.

### Identification and Quantification of Intracellular Organic Solute Pool by NMR Spectroscopy

All spectra were acquired on a Bruker AVANCE III 800 spectrometer (Bruker, Rheinstetten, Germany) working at a proton operating frequency of 800.33 MHz, equipped with a three channel 5 mm inverse detection probe head with pulse-field gradients along the Z axis.

^1^H-NMR spectra were acquired at 25°C using a 90° flip angle, with water presaturation during a relaxation delay of 2 s. ^13^C spectra were recorded at 201.24 MHz using the APT (attached proton test) sequence. Identification of the compounds was performed by comparison of ^1^H and ^13^C chemical shifts with the literature. For quantification purposes, ^1^H-NMR spectra were acquired with an extra delay of 60 s to allow complete signal relaxation and sodium formate was added as an internal concentration standard.

### The Effect of Salinity on Intracellular Organic Solute Pool

The effect of salinity in the accumulation of intracellular organic solutes was examined in cells grown in N^+^ medium (standard medium), in which ASW concentration varied from 50%, 73% (optimum salinity), 100%, 150% and 200% (vol/vol). These experiments were conducted during *R. baltica* exponential and stationary growth (turbidity at 610 nm, 1.2 and 1.7, respectively) at 25°C (optimum temperature). The extraction of compatible solutes was performed as described below. All experiments were performed in triplicate.

### The Effect of Nitrogen-depletion on the Organic Solute Pool

Nitrogen-depletion tests were performed using the standard medium designated N^+^ medium (1 g/L ammonium sulfate, standard condition) and under nitrogen-limiting conditions, designated N^−^ (medium without ammonium sulfate). Cells were grown at 25°C (optimum temperature) and 73% ASW (optimum salinity) in N^+^ medium until initial-exponential phase (OD_610_ = 0.6), harvested by centrifugation and re-suspended in an equal volume of N^+^ or N^−^ medium (optimum salinity), and incubated for 300 minutes at 25°C. After this period the extraction of compatible solutes was performed as described above. All experiments were performed in triplicate.

### The Effect of Thermal and Osmotic Shock on the Organic Solute Pool Under Nitrogen-limiting Conditions

In thermal shock tests, cells were continuously grown at 25°C (optimum temperature) in N^+^ medium, at the optimum salinity, until the initial-exponential phase (turbidity at 610 nm, 0.6), harvested by centrifugation and re-suspended in an equal volume of N^+^ (standard medium) or N^−^ medium (nitrogen-depleted medium), and incubated for 300 minutes at 20°C, 25°C and 35°C. After this period the extraction of compatible solutes was performed as described above. All experiments were performed in triplicate.

In osmotic shock experiments, cells were continuously grown at 25°C and optimum salinity in N^+^ medium until initial-exponential phase (turbidity at 610 nm, 0.6), collected by centrifugation and re-suspended in equal volumes of N^+^ or N^−^ media in which ASW concentration varied from 18%, 73%, 150% and 260% for 300 minutes. After this period the extraction of compatible solutes was performed as described above. All experiments were performed in triplicate.

## Results

### Identification and Quantification of the Organic Solute Pool in *R. baltica*

We first analyzed the compatible solute pool accumulated by *R. baltica* grown under standard conditions (25°C and medium containing 73% ASW) during the exponential and stationary phases by ^1^H-NMR spectra. This analysis allowed the identification of three major organic solutes, namely α-glutamate and lower amounts of sucrose and MGG (Fig. S1). The total solute pool was not dependent on the phase of growth under standard conditions; it remained stable at about 2.0 µmol/mg of protein during both exponential phase and stationary phase (Table 1). On the other hand, the proportion of the solutes accumulated depended on the phase of growth; α-glutamate was by far the major compatible solute present, with decreasing concentration from the exponential to the stationary phase (1.71±0.32 µmol/mg of protein to 1.35±0.27 µmol/mg of protein, respectively). Nevertheless, this amino-acid corresponded to more than 78% and 61% of the total solute pool accumulated by *R. baltica* during the exponential and stationary growth, respectively (Table 1). On the other hand, a notable increase in the levels of sucrose and MGG was observed in *R. baltica* cells, ranging from 7% (0.15 µmol/mg of protein) of the total solute pool during the exponential phase, for both sucrose and MGG, to 21,6% (0.48±0.03 µmol/mg of protein) and 14,4% (0.32±0.01 µmol/mg of protein) in the stationary phase, respectively (Table 1).

**Table 1:** Accumulation of compatible solutes in *R. baltica* grown at standard conditions.

Growth phase	Sucrose	MGG	α-Glutamate
Exponential	0.15±0.01	0.15±0.01	1.71±0.32
Stationary	0.48±0.03	0.32±0.01	1.35±0.27

Values are given in µmol/mg of protein.

Data are the means ± standard deviation of at least three measurements.

### Effect of Increasing Salinities on the Growth Rate and on the Organic Solute Pool

**Figure 1:**
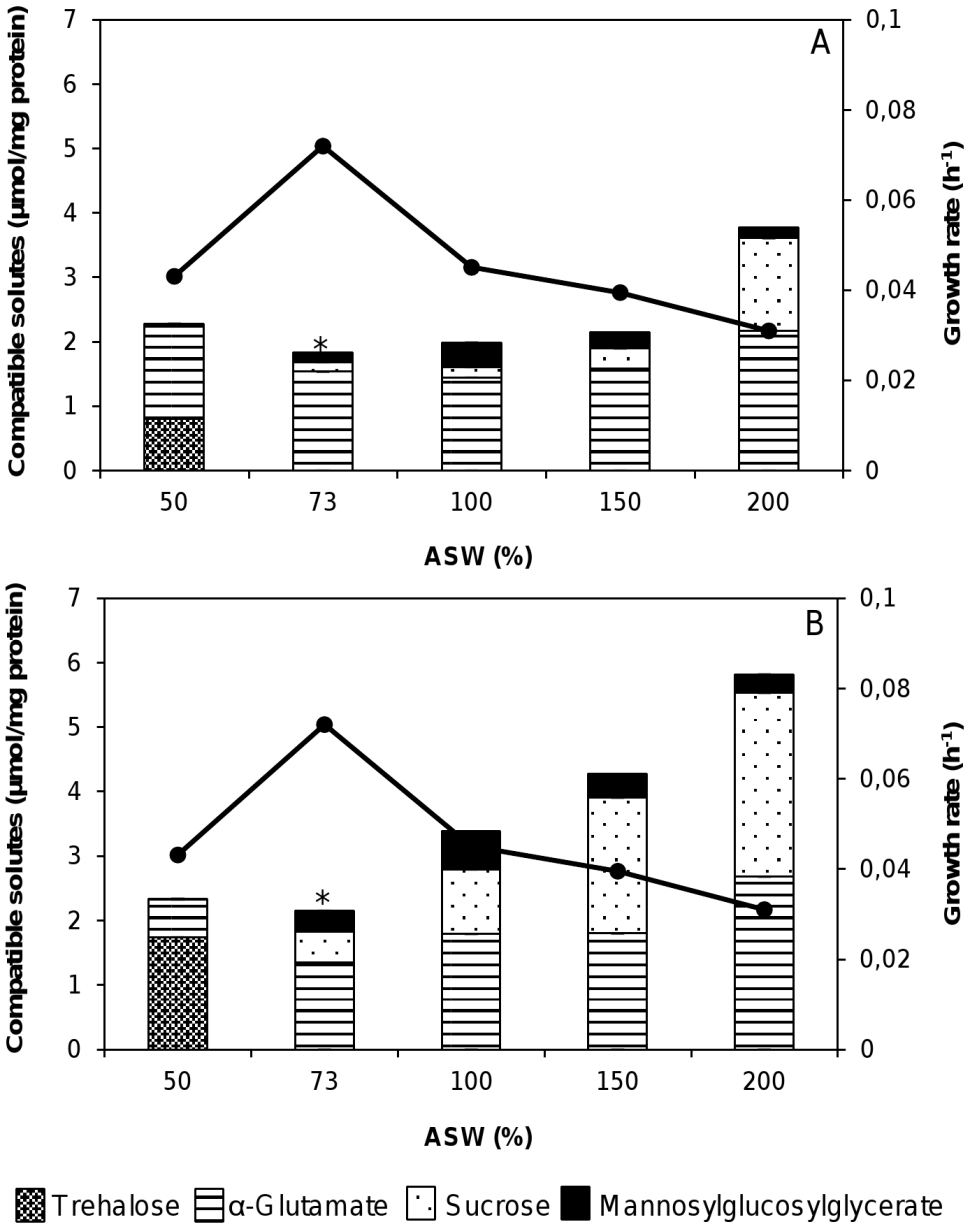
Effect of medium osmolarity. Influence of medium osmolarity on the growth rate of *R. baltica* (•) and on the accumulation of compatible solutes during the exponential phase (A) and the stationary phase (B). Bars represent intracellular concentrations. *, standard conditions. Data are the mean ± standard deviation of at least three measurements.

The optimum growth of *R. baltica* SH1^T^ occurred in medium containing 73% ASW. This organism was only able to grow within a narrow range of salinities. The organism did not grow in 18% ASW or in medium containing ASW above 200%. The dependence of the growth rate on the ASW concentration was typical of a slightly halophilic organism: the growth rate increased to some extent between 50% and 73% ASW, and decreased notably thereafter, at the same time as the salinity of the medium increased above the optimum conditions (Fig. 1).

The total solute pool under salt stress depended on the phase of growth and was higher in cells harvested during the stationary phase than in cells harvested during the exponential phase (Fig. 1). During the growth cycle there was a small but steady increase in α-glutamate and sucrose with increasing salinities, with a more pronounced effect in the stationary phase (Fig. 1B).

At the optimum salinity (73% ASW), α-glutamate was the preferred osmolyte of *R. baltica*, along with small levels of sucrose and MGG. Curiously, α-glutamate was the major organic solute under most of the conditions examined increasing slightly with salinity, except that trehalose and sucrose replaced α-glutamate in the stationary phase of growth in 50% and 200% ASW, respectively.

The organism responded to salt stress primarily by increasing the intracellular levels of sucrose. This trend was most notable at the highest salinity examined (200% ASW), since the level of sucrose represented 36 and 47% of the total organic solutes present in the exponential and stationary phase, respectively, showing a ten- and sixfold increase over those of the optimum growth conditions (Fig. 1).

### Trehalose as the Major Osmolytes Under Sub-optimal Salinities

Unexpectedly, a reduction in the salinity to sub-optimal conditions (50% ASW) led primarily to the accumulation of trehalose and α-glutamate, during both phases of growth. Moreover, sucrose was never detected under sub-optimal osmotic conditions. In cells grown in 50% ASW and harvested during the stationary phase, trehalose was by far the predominant compatible solute (1.74 µmol/mg of protein), representing 72% of the total organic solutes present. Nevertheless, α-glutamate also seemed to have a role in osmotic adjustment in 50% ASW, reaching values of 0.58 µmol/mg of protein (24% of total solute pool). However, this response was reversed in medium with higher ASW concentrations, since sucrose replaced trehalose as the most abundant sugar-derivative compatible solute, whereas trehalose became undetectable (Fig. 1).

### Effect of Nitrogen-depletion on the Organic Solutes Pool

α-Glutamate, the preferred osmolyte of *R. baltica* grown under standard nitrogen conditions (1 g/L ammonium sulfate, N^+^ medium), needs both nitrogen and carbon for its biosynthesis, whereas the sugar-derivative compatible solutes needs carbon but not nitrogen. The C/N ratio of the growth medium could affect the balance between glutamate and sugar-derived compatible solutes. Since marine bacteria growth is usually limited by the amount of nitrogen available in the open ocean [17], we considered it important to investigate the role of nitrogen-depletion on α-glutamate content and simultaneously on the levels of the other osmolytes. We therefore, induced a sudden change on the nitrogen concentration under optimum salinity and temperature (73% ASW and 25°C) and measured the stress response of *R. baltica* for a limited period of time at the compatible solute pool level.

**Figure 2:**
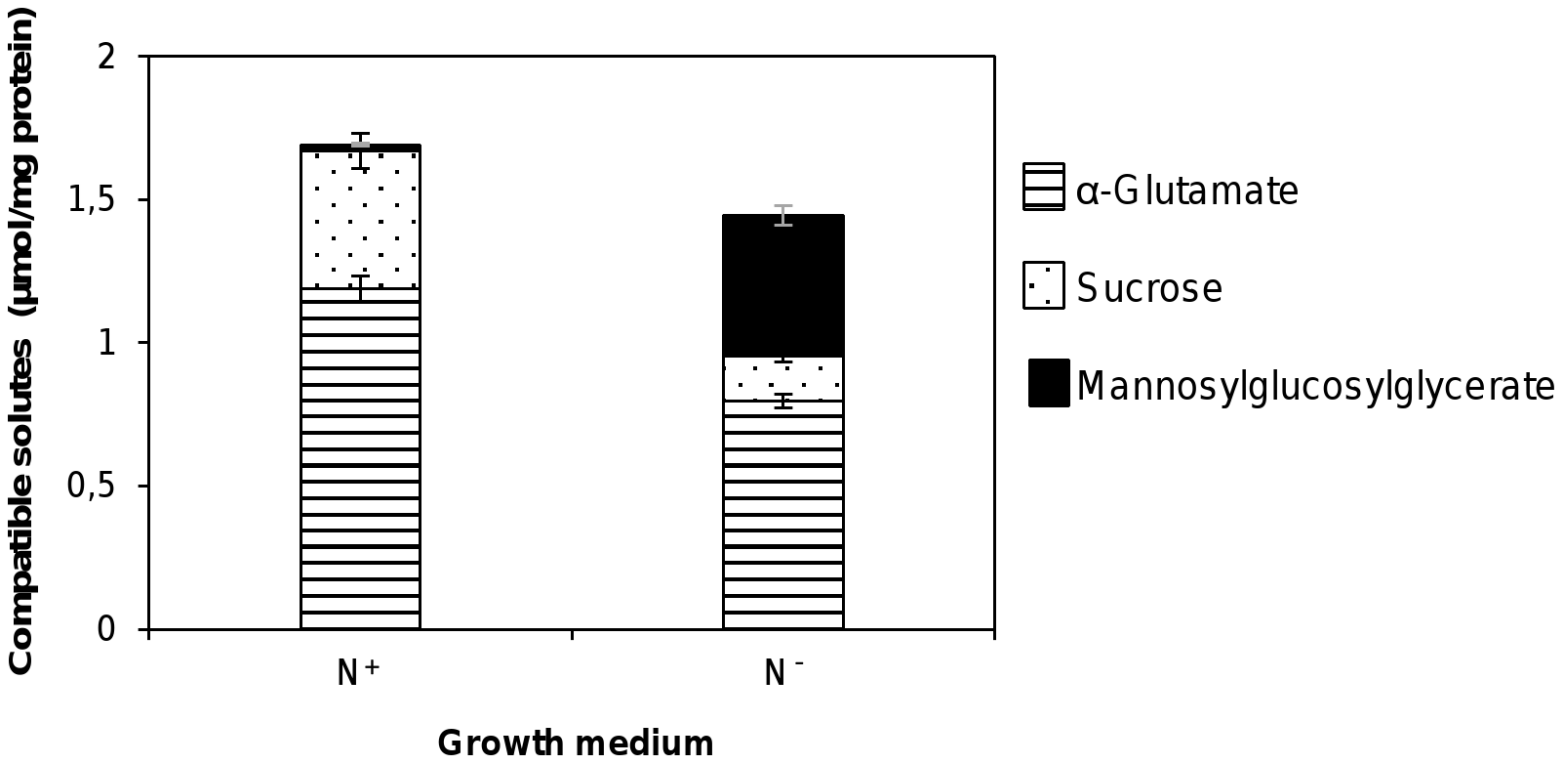
Accumulation of compatible solutes in *R. baltica* grown under standard and nitrogen-limiting conditions. Accumulation of compatible solutes in *R. baltica* grown under standard conditions (N^+^) and nitrogen-limiting conditions (N^−^) after 300 minutes incubation. Bars represent intracellular concentrations. Data are the mean ± standard deviation of at least three measurements.

Resuspending cells in nitrogen-depleted medium reduced the total amount of osmolytes by about 10%. The compatible solute pool composition in standard and nitrogen-limiting conditions was distinct indicating a dependency on nitrogen availability (Fig. 2). The most obvious result was a 25 fold increase in MGG levels under nitrogen-limiting conditions. This clear rise of MGG concentration was accompanied by a dramatic decrease in sucrose levels in nitrogen-depleted medium. The α-glutamate content also decreased in N-depleted conditions, but this amino acid continued to be the preferred compatible solute accumulated under all conditions examined (Fig. 2).

### Effect of Osmotic Shock on the Organic Solute Pool Under Nitrogen-limiting Conditions

As mentioned above, a remarkable increase in MGG levels was observed in nitrogen-limiting conditions under optimum salinity in contrast to the very low level accumulated in standard conditions. We also tested the effect of a combined nitrogen-limitation and osmotic shock on compatible solute accumulation.

**Figure 3:**
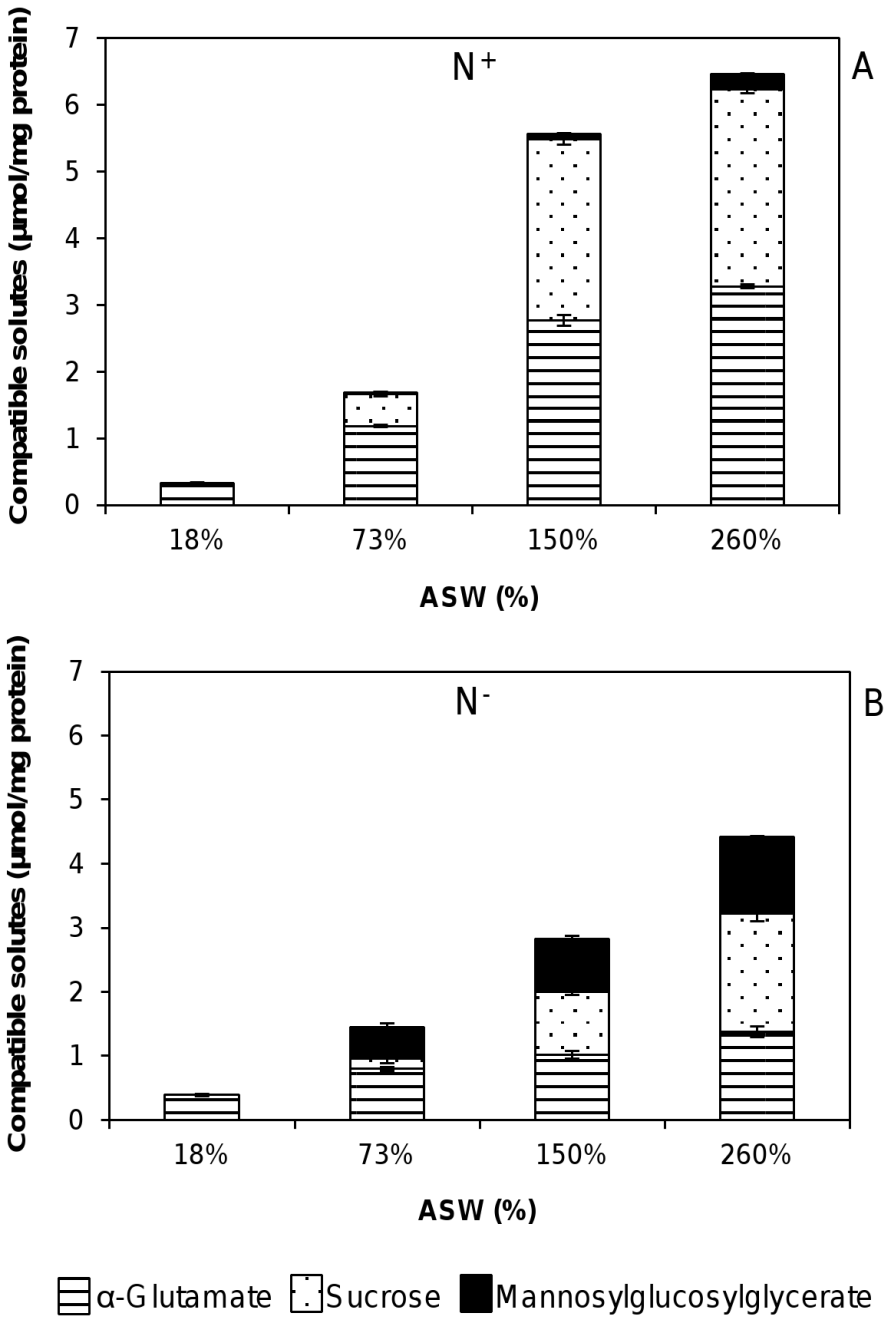
Effect of salinity and nitrogen-depletion on the accumulation of compatible solutes in *R. baltica*. Effect of salinity and nitrogen-depletion on the accumulation of compatible solutes in *R. baltica* under standard conditions (A, N^+^) and nitrogen-limiting conditions (B, N^−^). Bars represent intracellular concentrations. Data are the mean ± standard deviation of at least three measurements.

As expected, the total internal solute pool was dependent on the nitrogen availability as well as on the salt levels. The osmotic adaptation of the cells during short-term osmotic stress in N^+^ or N^−^ conditions led to an increase of the total amount of compatible solutes with higher ASW under both conditions. This trend was most notable at the highest salinity examined (260% ASW), leading to a three- and four-fold increase in the levels of compatible solutes over those of the optimum osmotic conditions, in N^−^ and N^+^ conditions, respectively (Fig. 3). Eliminating nitrogen from the medium under optimum osmotic conditions (73% ASW) resulted in the reduction of the total amount of osmolytes by about 10%. In addition, a dramatic decrease in the osmolyte content was observed when the salinity was increased under nitrogen-limiting conditions as compared to standard nitrogen conditions (Fig. 3). This tendency was most notable at the highest salinity examined (260% ASW), since the total amount of solutes accumulated in N^−^ medium was about two times lower than under standard (4,6 µmol/mg of protein and 7,5 µmol/mg of protein, respectively).

Additionally, the combination of nitrogen-limiting conditions and osmotic stress in *R. baltica* led to a shift of the pattern of the compatible solutes accumulated. In medium containing nitrogen, *R. baltica* responded to sudden salt stress primarily by increasing the intracellular levels of sucrose, representing 47 and 39% of the total organic solutes present in medium containing 150% and 260% ASW, respectively (Fig. 3). Likewise, the concentration of α-glutamate also increased with salinity, from 1.19±0.02 µmol/mg of protein in 73% ASW (optimum conditions) to 3.28±0.03 µmol/mg of protein in 260% ASW. Nevertheless, its relative proportion decreased with the salinity of the medium, representing approximately 65, 48 and 44% of the total osmolytes at 73, 150 and 260% ASW, respectively. The osmotic shock also led to a small, but steady increase of MGG levels under N^+^ conditions, corresponding to 3% of the total organic solute pool at 260% ASW (Fig. 3A).

The elimination of the nitrogen source from the medium caused a profound impact on the pattern and relative concentration of osmolytes. Accordingly, a dramatic decrease of α-glutamate levels was observed when compared to N^+^ conditions, under all tested salinities. The expected decrease in α-glutamate content was accompanied by a very large increase of MGG levels. Indeed, MGG reached 26% of the total organic solute pool at 260% ASW, corresponding to 1.2 µmol/mg of protein versus 0.22 µmol/mg of protein in nitrogen-rich medium. In fact, MGG levels clearly increased in salt-loaded cells grown under N-limiting conditions, while the internal α-glutamate and sucrose levels decreased.

On the other hand, the solute pool accumulated by *R. baltica* subjected to hypo-osmotic shock (18% ASW) comprised only α-glutamate, being low under N^+^ and N^−^ conditions when compared (4.8 and 3.4 fold decrease, respectively) to the optimum salinity (73%).

### Effect of Thermal Shock on the Organic Solute Pool Under Nitrogen-limiting Conditions

We also examined the effect of thermal shock under nitrogen-limiting conditions on solute accumulation, by inducing a sudden change in the growth temperature under N^+^ and N^−^ medium and characterizing the pool of compatible solute accumulated by *R. baltica*.

**Figure 4:**
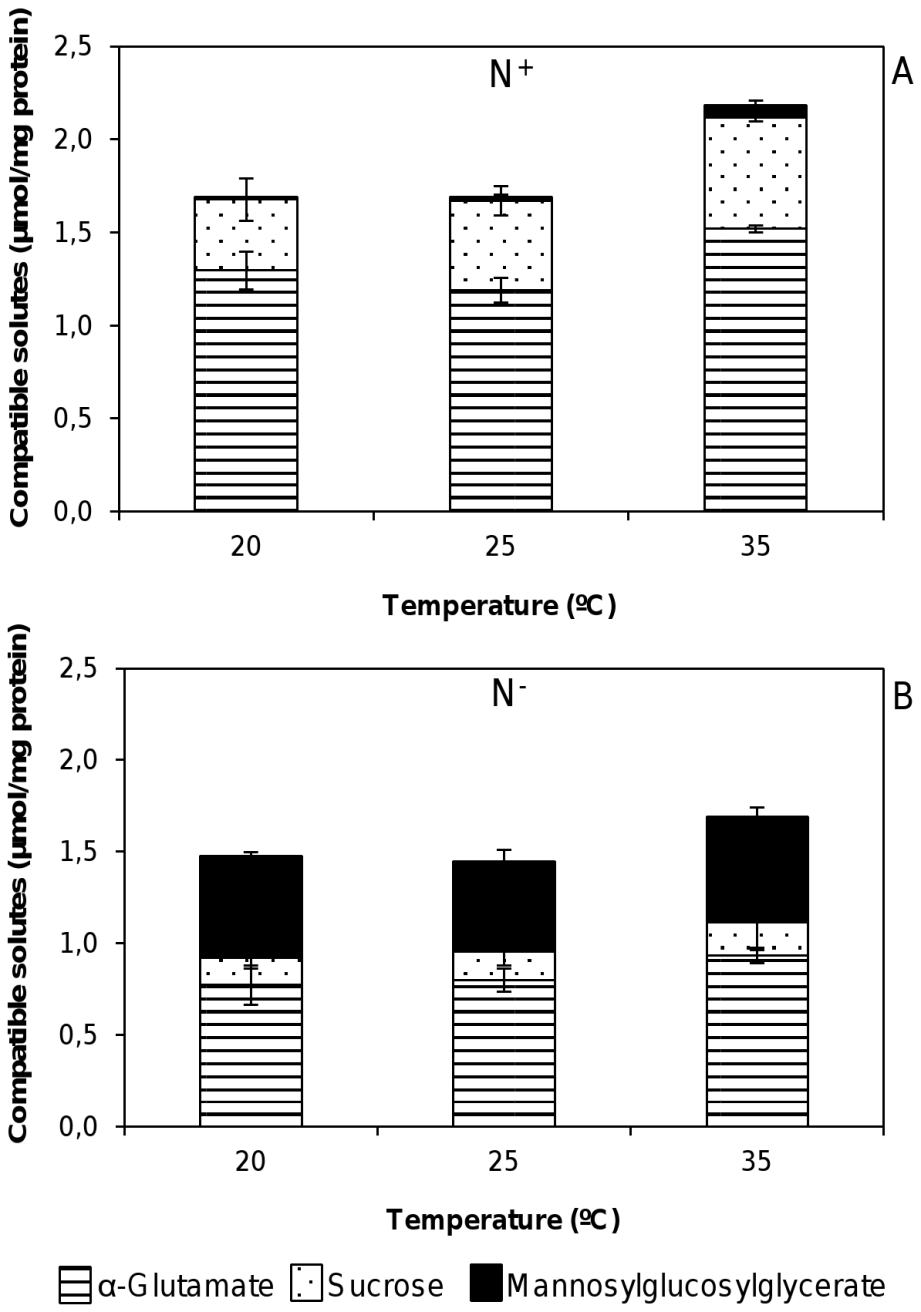
Effect of temperature and nitrogen-depletion on the accumulation of compatible solutes in *R.* baltica. Effect of temperature and nitrogen-depletion on the accumulation of compatible solutes in *R. baltica* under standard conditions (A, N^+^) and nitrogen-limiting conditions (B, N^−^). Bars represent intracellular concentrations. Data are the mean ± standard deviation of at least three measurements.

An overall analysis showed that the total internal compatible solute pool varied little with temperature but was dependent on the nitrogen availability. The thermal shock below the optimum temperature (25°C) under standard nitrogen conditions did not have any significant effect on the compatible solutes accumulated by *R. baltica*. Meanwhile, thermal shock experiments at supra-optimal temperature (35°C) resulted in a slight increase in the accumulation of α-glutamate, from 1.19±0.06 µmol/mg of protein to 1.52±0.02 µmol/mg of protein, along with minor levels of MGG, from 0.02±0.01 µmol/mg of protein to 0.06±0.03 µmol/mg of protein (Fig. 4A).

Once again the most important effect was caused by the depletion of nitrogen in the medium. Regardless of the temperature tested, under N^−^ conditions when compared with N^+^ conditions, a decrease in the levels of α-glutamate and sucrose was observed coupled with a remarkable increase in MGG levels (Fig. 4). Indeed, a tenfold increase of MGG was observed at the highest thermal shock examined (35°C), corresponding to 0.58 µmol/mg of protein of the total solute. On the other hand, it was observed a twofold decrease of α-glutamate levels, corresponding to 1.52 µmol/mg of protein versus 0.93 µmol/mg of protein, in N^+^ medium and N^−^ medium, respectively. Moreover, as expected it was also observed a threefold lower content of sucrose in N^−^ conditions when compared to standard nitrogen conditions (Fig. 4).

## Discussion

Environmental changes can induce different stress responses on marine organisms. This work helps to understand how *R. baltica* can adapt not only to osmotic stress but also to nitrogen-depletion, which is also a limiting resource in this environment. We tested different types of stresses on *R. baltica* namely, nitrogen-depletion, thermal and osmotic shock under nitrogen-limiting conditions to characterize the osmoadaptation strategy used by this organism.

The total solute pool under salt stress depended on the phase of growth and it was higher in cells harvested during the stationary phase than in cells harvested during the exponential phase. One can assume a dual function for the compatible solutes accumulated during the stationary phase, since they can act as osmolytes and also function as carbon and nitrogen reserves (1–4).

The analysis of the osmolyte content of *R. baltica* cells revealed the accumulation of α-glutamate, sucrose, trehalose and MGG. The ratio and total intracellular organic solutes varied significantly in response to an increase in salinity. α-Glutamate was definitely the preferred osmolyte accumulated by *R. baltica* during osmotic and thermal adaptation under most conditions examined. Many mesophilic bacteria and archaea challenged with increased external salinity display a preliminary response that includes α-glutamate synthesis up to the start point of a more pronounced osmoadaptation mechanism [3], [4]. Our results are in agreement with this tendency since the increase on α-glutamate levels in the exponential phase was partially replaced by an increase of sucrose and MGG levels in the stationary phase. Nevertheless, glutamate (α- and β-form) occurs frequently in hyper/thermophilic archaea and bacteria for osmoadaptation under salt stress (*Archaeoglobus fulgidus*, *Thermococcus litoralis*, *Aquifex pyrophilus*, *Palaeococcus ferrophilus*, *Petrotoga miotherma* and *Methanotorris igneus*) [9], [25]. Therefore, the observation that *R. baltica* salt stress response also involves the accumulation of α-glutamate is clearly in line with this trend.

Interestingly, along with α-glutamate, trehalose becomes a major solute accumulated but only at low medium osmolarity, whereas sucrose, along with α-glutamate, are the preferred compatible solutes under all other osmotic stress conditions. Indeed, the switch between trehalose and sucrose was certainly the most significant effect caused by salinity changes of the medium in terms of compatible solute accumulation in *R. baltica*. Generally, the accumulation of sucrose or trehalose as main compatible solutes is associated, in freshwater cyanobacteria, with a low salt tolerance. Indeed, the highest salt tolerance for sucrose-accumulating cyanobacteria comprises salinities equivalent to 50–100% seawater [7]. Like cyanobacteria, *R. baltica* relies mostly on the accumulation of trehalose to cope with low osmotic conditions. In contrast, *R. baltica* responded to salt stress mostly by increasing the intracellular levels of sucrose, and to a minor extent of α-glutamate. This trend was most perceptible at the highest salinity examined (200% ASW), representing a six fold increase of sucrose levels over those accumulated during optimum growth conditions. Similarly, sucrose was recently identified as a main compatible solute in a marine *Prochlorococcus* sp. under osmotic stress conditions [17], refuting the idea that sucrose accumulation does not supports growth in full-strength marine media [26].

Bacteria are able to synthesize several osmolytes and adapt their relative ratios to medium composition. Indeed, the ratio of *R. baltica* osmolytes varied with medium composition. In standard nitrogen media, MGG constitutes a minor solute whereas a spectacular increase of MGG concentration occurs under nitrogen-limiting conditions with increasing salinity. Correspondingly, the depletion of nitrogen in the medium produced a dramatic decrease in the levels of α-glutamate. From our results, we can foresee a dual role for MGG in *R. baltica*, it replaces α-glutamate as a compatible solute and acts as a counterion for K^+^ in salt-stressed cells to maintain electroneutrality. It could be also hypothesized that MGG is more efficient in osmoregulation when compared with α-glutamate and sucrose, explaining the observed decreased on the total solute pool concentration under nitrogen-limiting conditions.

The accumulation of MGG by *R. baltica* came somewhat as a surprise, since MGG has only been described in two thermophilic strains of the genus *Petrotoga* [8], [9] and so far was not known to have a role in osmotic adaptation in mesophilic bacteria. MGG accumulates in *P. miotherma* during low-level osmotic adaptation whereas in *P. mobilis* responds to hyperosmotic conditions and supra-optimal growth temperatures [8], [9]. The presence of this solute in the endogenous osmolyte pool of *R. baltica* is interesting because this bacterium is phylogenetically distinct from these thermophilic strains. Nevertheless, in *R. baltica*, MGG responds as a classical compatible solute under specific nutritional constrains since its accumulation increased proportionally with the medium salinity under nitrogen-limiting conditions. This stimulatory effect caused by a combination of two stress conditions has already been reported, in several organisms where the ratios of the several compatible solutes accumulated were also highly dependent on the growth substrate. In *Dickeya dadantii* strain 3937 (former *Erwinia chrysanthemi* 3937) the preferred compatible solutes during salt stress were glutamine, α-glutamate and lower levels of glucosylglycerate (GG). However, maximal reduction of nitrogen concentration in salt-containing medium resulted in a rapid decline of glutamine and α-glutamate concentrations to almost undetectable levels, together with their replacement by GG, becoming the main and even single compatible solute [27]. Undoubtedly, these results demonstrated that without a nitrogen source for the synthesis of nitrogen-rich amino acid compatible solutes, *Dickeya dadantii* synthesizes and accumulates a nitrogen free compatible solute, GG. Similar results were observed for the slightly halophilic cyanobacteria *Prochlorococcus marinus* and *Synechococcus* sp. PCC7002 [17]. The typical compatible solute of marine cyanobacteria is the neutral glucosylglycerol but it is absent from the above mentioned organisms. Klähn and colleagues (2010) showed that GG levels increased with the salinity of the medium, especially under nitrogen-limiting conditions. In these organisms, GG replaces α-glutamate as the counter ion for K^+^ during salt stress and nitrogen depletion. These observations were the proof for a tight regulation between salt stress and nitrogen deficiency.

The finding that the marine bacterium *R. baltica* accumulates MGG and sucrose leads us to speculate that the range of conditions for the accumulation of these compatible solutes is more flexible than initially suspected.

## Supporting Information

Figure S1 ***R. baltica*** **extract proton spectrum.** Proton spectrum (A) and sugar anomeric region of the proton spectrum (B) acquired at 800.33 MHz of an extract of *R. baltica* grown at 25°C with 150% ASW in N^+^ medium.
(PDF)
