# Supplementary material for: A Unique Pool of Compatible Solutes on Rhodopirellula baltica, Member of the Deep-Branching Phylum Planctomycetes
Source: PLoS One. 2013 Jun 27;8(6):e68289. doi: 10.1371/journal.pone.0068289 (PMC3694870; doi:10.1371/journal.pone.0068289)
Supplement: Figure S1 — R. baltica extract proton spectrum. Proton spectrum (A) and sugar anomeric region of the proton spectrum (B) acquired at 800.33 MHz of an extract of R. baltica grown at 25°C with 150% ASW in N+ medium. (PDF) [file pone.0068289.s001.pdf]

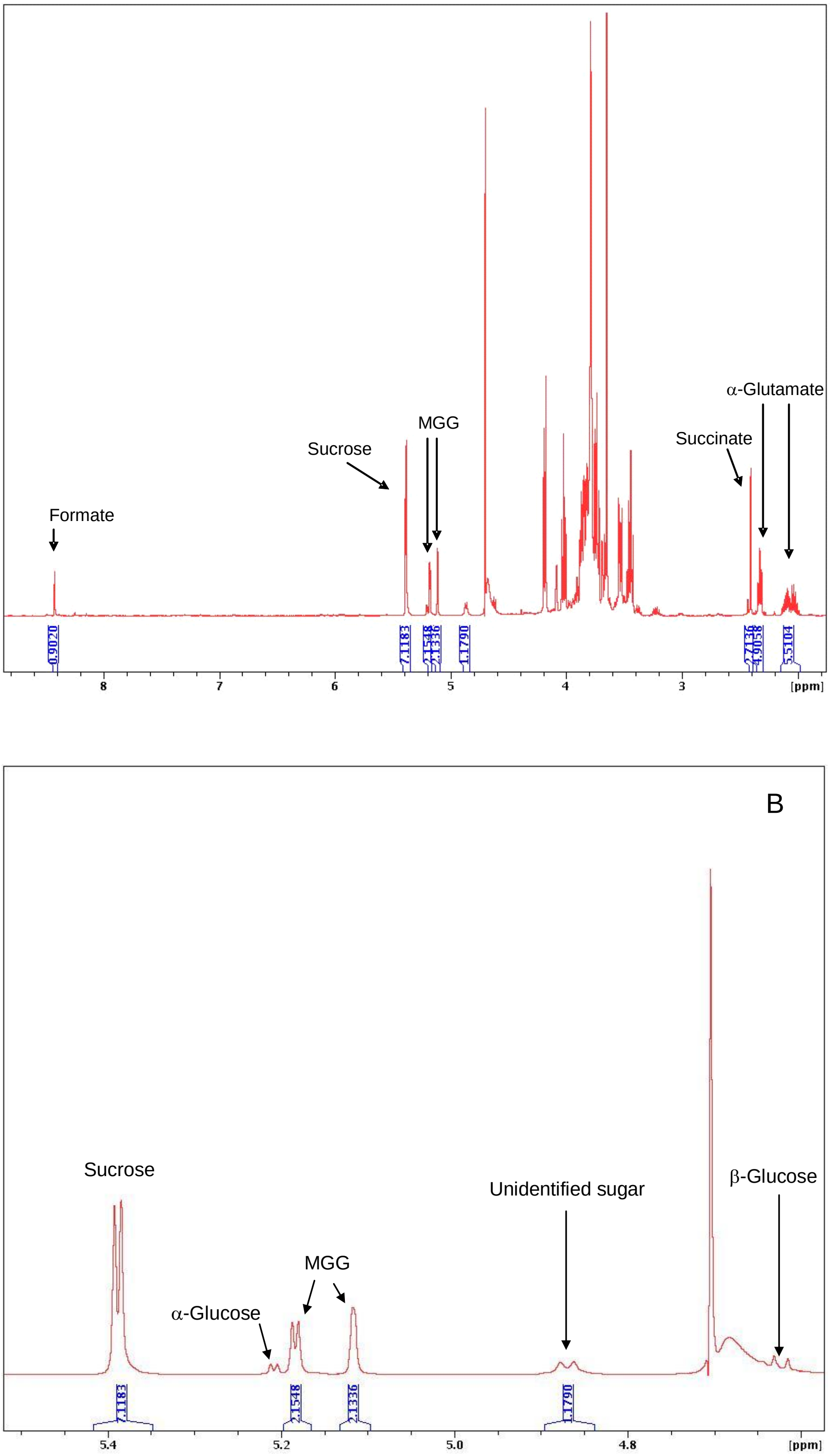

Figure S1. *R. baltica* extract proton spectrum. Proton spectrum (A) and sugar anomeric region of the proton spectrum (B) acquired at 800.33 MHz of an extract of *R. baltica* grown at 25ºC with 150% ASW in N^+ medium.
